# Conference of State Boards of Health

**Published:** 1888-06

**Authors:** 


### CONFERENCE OF STATE BOARDS OF HEALTH.

A conference of State Boards of Health, was held in Cincinnati in May, composed of one or more delegates from each of the State Boards of Health in the United States. Dr. J. N. McCormack, of Bowling Green, Ky., presided, and Dr. C. O. Probst, of Ohio, acted as Secretary.

Among the physicians present were Drs. John D. Jones, N. P. Dandridge, William Carson, of Cincinnati; H. S. Orme, California; R. S. Goodwin, Connecticut; J. H. Rauch, Illinois; S. R. Searight, W. A. Fritsch, S. S. Roots, J. M. Taylor, Indiana; Pinckney Thompson, Kentucky; J. J. N. McCormack, Kentucky; R. W. Llewelyn, Iowa; W. S. Schenk, Kansas; Welch, Kansas; H. B. Baker, Michigan; C. N. Hewitt, Minnesota; C. O. Probst, Ohio; Benjamin Lee, Philadelphia; David Engelman, Pennsylvania; James Evans, South Carolina; J. T. Reeve, Wisconsin; B. O. Reynolds, Wisconsin, and P. H. Bryce, Province of Ontario, Canada.

Dr. McCormack, the President of the association, delivered the annual address in which he mentioned the fact, so well known, that the quarantine facilities of New York are totally inadequate to the demands of the service. The topic for discussion was then read: "Duties of the Conference in urging the erection of isolated hospitals for infectious diseases, and economical and effective methods of placarding diseased houses and quarantining diseased families."

Dr. BRYCE, of Ontario, read a brief paper upon the subject. He urged the importance of the isolation of all cases of diphtheria, scarlatina, and other infectious diseases of that type, and stated that the only means by which the necessary isolation could be obtained was by the establishment and maintenance of hospitals of that nature by townships and municipalities. When proper isolation could be obtained in private residences removal to a hospital was unnecessary, but as a general thing such isolation was not to be had, especially among the poor people of the large cities.

Dr. PROBST believed that isolated hospitals for the treatment of infectious diseases would be a good thing, but he doubted its practicability. Dr. Taylor, of Indiana, coincided with Dr. Bryce as to the results to be obtained by his plan, but doubted its feasibility. It might be practicable in very large cities where hospital conveniences were abundant, but in small towns and sparsely settled districts it would be impracticable, and the effect of removal over long distances dangerous. Dr. Thompson, of Kentucky, supported Dr. Taylor. Dr. Lee, of Philadelphia, agreed with Dr. Bryce in his conclusions as to the practicability of the plan proposed, and so did Dr. Hewitt, of Minnesota. Dr. H. S. Orme, of California, also advocated the plan. The Chair appointed Dr. Orme, Dr. Hewitt, and Dr. Bryce as a committee to investigate the subject and report later.

"Should the National Government assume the control of quarantines at all ports of entry?" "Under which control should quarantine be both in Canada and the Union, under National Government or under State governments?" were questions that occupied the time of the sanitarians at the next session. Dr. Lee, of Philadelphia, advocated strict regulation and control by the Government of all ports on the coasts where it was at all possible that infectious diseases could be imported, and offered the following resolution, which was referred to the regular standing committee:

*Resolved*, That this Conference, recognizing the failure of local authorities to administer quarantine effectually in a large number of cases, respectfully urges upon the National Government the duty of assuming the control of quarantine at all ports of entry.

Dr. HEWITT said, in reference to the resolution, that it was impossible to look to Congress for action in the premises; that no appropriation was available for adequate quarantine protection, and that if anything was to be done it must be done separately by the State Boards of Health.

Dr. RAUCH agreed with Dr. Hewitt as to the inability of obtaining the necessary national legislation for quarantining the coasts. He was, of course, in favor of a national system of protective quarantine from pestilential diseases, but he was opposed to putting such a system, even if obtainable, into the hands of the U. S. Marine Hospital authorities.

Dr. BAKER said he had no confidence in the efficiency or practicability of a national system of coast quarantine. He favored the continuance of action on the part of the State governments, and the obtaining of national aid in emergencies therefor if it were possible. In support of his statement he quoted the well known—to physicians–efficiency of the New Orleans quarantine station, which is under the control of the local authorities, an efficiency which, he said, would be weakened were the national authorities to be placed in control.

The New York quarantine service was notably inefficient, but if the proper efforts were made the service would be brought up to the degree of efficiency of the New Orleans stations. Dr. Baker sarcastically said that he did not believe there was a skilled sanitarian in all the National Marine Hospital service.

Dr. KENNEDY stated that he was not at all impressed with the value of the inland quarantine system, and said that he feared an epidemic of yellow fever and cholera this year, but he did not state the basis of his fears, though he intimated that they were due to lack of an effective system of quarantine.

Dr. BRYCE described the workings of the Canadian quarantine system.

Dr. HEWITT asked the Chair to appoint a committee to confer and correspond with and visit the maritime quarantine authorities and to investigate and ascertain the best methods of quarantining from infectious diseases.

The question proposed by the State Board of Health of Vermont, "What legal authority ought State Boards of Health to possess in the absence of local boards?" was widely discussed by the Conference, the discussion consisting of a statement of the laws and systems in effect and in use in the different States.

Dr. HEWITT, of Minnesota, stated the vigorous measures used in that State to suppress and stamp out infectious dieases. He said that in Minneapolis there occured a small epidemic of seventy cases of smallpox. The board took immediate action, and each house was thoroughly disinfected, the patient removed, and every one who had been in the house for a week was traced and quarantined and disinfected before the disease had a chance to spread.

At the evening session the following questions, proposed by the Michigan Board, were discussed:

"What is each State Board of Health doing to advance sanitary science?"

"By the collection of statistics of deaths, and their causes."

"By the collection of statistics of sickness."

"By the collection of statistics of meteorogical conditions coincident with sickness and deaths."

The physicians present stated the system and efficiency of the collection of vital statistics in the different States represented in the Conference.

